# Isolation, Identification, and Function of *Rhodotorula mucilaginosa* TZR_2014_ and Its Effects on the Growth and Health of Weaned Piglets

**DOI:** 10.3389/fmicb.2022.922136

**Published:** 2022-07-12

**Authors:** Ping Hu, Junxia Mao, Yan Zeng, Zhihong Sun, Huan Deng, Chen Chen, Weizhong Sun, Zhiru Tang

**Affiliations:** ^1^Laboratory for Bio-Feed and Animal Nutrition, Chongqing Key Laboratory of Herbivore Science, College of Animal Science and Technology, Southwest University, Chongqing, China; ^2^Fermentation Engineering Department, Hunan Institute of Microbiology, Changsha, China

**Keywords:** *Rhodotorula mucilaginosa* TZR_2014_, isolation and identification, intestinal health, antioxidant capacity, weaned piglets

## Abstract

A red yeast isolated from orange and grape soil and identified by the 26S rDNA sequence analysis revealed that it was *Rhodotorula mucilaginosa* and named TZR_2014_. Its biomass and carotenoid production reached a maximum when using the fermentation medium with pH 6.0, containing 5% glucose, 1% peptone, and 1.5% yeast powder. TZR_2014_ was resistant to 55°C for 15 min, 0.2% pig bile salts for 4 h, and artificial gastric and intestinal fluids. A total of thirty 28-day weaned pigs were divided into three groups, and the piglets were fed a basal diet (CON), a basal diet and orally administered 1 ml 1.0 × 10^10^ CFU/ml *Candida utilis* DSM 2361 three times (*C. utilis*), or a basal diet and orally administered 1 ml 1.0 × 10^10^ CFU/mL TZR_2014_ three times daily (*R. mucilaginosa*) for 4 weeks. Compared with the piglets in the CON group, those in the *C. utilis* or *R. mucilaginosa* group reported an increased average daily weight gain and average daily feed intake (*P* < 0.05) and a decreased feed/gain (*P* < 0.05). The diarrhea rate of piglets in the *R. mucilaginosa* group was lower than that in the CON and *C. utilis* groups (*P* < 0.05). Compared with that in the CON and *C. utilis* groups, the *R. mucilaginosa* group reported an increased ileum villus height (*P* < 0.05), serum concentration of total antioxidant content, total superoxide dismutase, and glutathione peroxidase and pepsin and lipase activities in the intestinal content, while it reported a decreased serum concentration of malondialdehyde and pH of the intestinal tract (*P* < 0.05). The relative abundances of *Proteobacteria* and *Megasphaera* of caecum in the *R. mucilaginosa* group were lower than those in the CON and *C. utilis* group*s* (*P* < 0.05). The relative abundances of *Prevotella, Ruminococcaceae, Succinivibrio, Rikenellaceae RC9 gut group*, and *Roseburia* of caecum in the *R. mucilaginosa* group were higher than those in the CON and *C. utilis* group*s* (*P* < 0.05). *R. mucilaginosa* TZR_2014_ can produce carotenoids and adapts to the animal's gastrointestinal environment. Oral *R. mucilaginosa* TZR_2014_ improved growth performance, enhanced antioxidant capacity, strengthened gastrointestinal digestion, and maintained the intestinal microbiological balance of piglets.

## Introduction

Based on the concept of healthy feeding, probiotics not only help avoid the adverse effects of antibiotics on weaned piglets but also promote intestinal health. Yeast is a common, safe microecological agent. As a saprophytic microbe, *Rhodotorula* widely exists in the soil, sea, animals, and plants. *Rhodotorula mucilaginosa* has high nutritional value and probiotic effects (Nelis and De Leenheer, 1991). Glucan and mannan in the cell wall of *R. mucilaginosa* can enhance the migration and phagocytosis of macrophages and neutrophils, reduce intestinal inflammatory reactions, enhance animal resistance, promote the reproduction of beneficial bacteria, and competitively inhibit the colonization of harmful bacteria (Sakai, 1999; Dalmo and Bogwald, 2008). *R. mucilaginosa* products contain many carotenoids and zymochromes (Aksu and Eren, 2005); previous studies have shown that carotenoids are beneficial to human and animal health (Mannazzu et al., 2015). As the precursor of vitamin A, carotenoids can resist cancer, inhibit gene mutations, and resist the side effects of environment-induced genotoxic agents by regulating cell signaling and gene expression. Carotenoids are also recognized as super antioxidants (Dalmo and Bogwald, 2008; Bhagavathy and Sumathi, 2012), which have various health functions, such as enhancing host immunity, demonstrating anti-oxidation and anti-tumor activity, and lowering blood pressure (Sharma and Ghoshal, 2020). However, there are few studies on the application of *R. mucilaginosa* in livestock production, and its safety has not yet been verified. In this study, *R. mucilaginosa* TZR_2014_ was isolated and identified from orchard soil, and its safety was tested to study the effects of *R. mucilaginosa* TZR_2014_ on weaned piglets.

## Materials and Methods

### Isolation, Identification, and Physiological and Biochemical Characteristics of *R. mucilaginosa TZR_2014_*

#### Microorganisms, Culture Media, and Reagents

Soil samples containing *R. mucilaginosa* TZR_2014_ were collected from the Jingguoyuan orchard (Jingyun Mountain, Chongqing, China) on 8 September 2014 (Tang et al., 2016). Jingguoyuan is a fruit garden located at 500 m a.s.l. on the east side of the Jingyun Mountain, Chongqing, China, where the summer and winter temperatures are ~32 and 10°C, respectively. The enrichment culture medium comprised 0.1% (w/v) urea, 5% (w/v) glucose, 0.05% (w/v) yeast extract, 0.1% (w/v) (NH_4_)_2_SO_4_, 0.1% (w/v) MgSO_4_ 7H_2_O, 0.25% (w/v) KH_2_PO_4_, 0.01% (w/v) FeSO_4_ 7H_2_O, and 0.003% (w/v) Bangladesh Red, adjusted to pH 4.5, and sterilized for 15 min at 115°C. The potato dextrose agar (PDA) medium comprised 200 g of potato, 20 g of glucose, 20 g of agar powder, and distilled water. A constant volume of 1 L was maintained at natural pH and sterilized for 15 min at 115°C. The yeast extract peptone dextrose (YPD) culture medium comprised 2% (w/v) glucose, 1% (w/v) yeast powder, 2% (w/v) peptone, and 2% (w/v) agar, adjusted to pH 6.0, and sterilized for 15 min at 115°C. Nutrient broth (NB) medium comprised 0.3% (w/v) beef extract, 0.5% (w/v) NaCl, and 1% (w/v) peptone, adjusted to pH 7.0, and sterilized for 20 min at 121°C. The seed culture medium comprised 1% (w/v) peptone, 2% (w/v) glucose, and 1% (w/v) yeast powder, adjusted to pH 6.0, and sterilized for 15 min at 115°C. The fermentation medium comprised 1% peptone (w/v), 5% glucose (w/v), and 1.5% yeast powder (w/v), adjusted to pH 6.0, and sterilized for 15 min at 115°C.

#### Isolation and Morphological, Physiological, and Biochemical Characterization

One gram of soil containing yeast was mixed with 100 ml of yeast liquid enrichment medium. The suspension (1 ml) was serially diluted 10 times to 10^−6^. The 100 μl suspension from the diluted samples was plated on PDA solid medium and cultured at 28°C for 2–3 days. Three-day-old cultures on PDA agar were used for cellular and colony morphology analyses of yeast. The morphology of *R. mucilaginosa* cells was photographed using an Olympus BX40 microscope (Olympus, Tokyo, Japan). Routine physiological and biochemical tests were conducted according to the characteristics and identification described by Barnett et al. (1983).

#### 26S rDNA D1/D2 Identification of *R. mucilaginosa* TZR_2014_

Three-day-old cultures of yeast in PDA liquid medium were collected and directly used for DNA extraction, according to the instructions of the Power DNA Isolation Kit (Mobio, Carlsbad, CA, USA). DNA was quantified using a Nanodrop spectrophotometre (Nyxor Biotech, Paris, France), followed by staining with the Quant Pico Green dsDNA Kit (Invitrogen Ltd., Paisley, UK). PCR amplification of the D1/D2 region of yeast 26S rDNA was performed using the universal primers NL1 (5'-GCATATCAATAAGCG GAGGAAAAG-3') and NL4 (5'-GGTCCGTGTT TCAAGACGG-3'; Thanh et al., 2002). The cycling parameters were as follows: initial denaturation at 94°C for 4 min, 25 cycles of denaturation at 94°C (40 s), annealing at 55°C (45 s), elongation at 72°C (30 s), and final extension at 72°C for 10 min. PCR reaction system consisted of the following: DNA template (20–50 ng/μl) 0.5 μl; 10 × buffer (with Mg^2+^) 2.5 μl; dNTP (2.5 mM) 1 μl; Taq polymerase 0.2 μl; primer F (10 μM) 0.5 μl; primer R (10 μM) 0.5 μl; and ddH_2_O 25 μl. The PCR products were separated by 1.0% agarose gel electrophoresis (150 V, 100 mA, 20 min) and purified using a QIAquick Gel Extraction Kit (Qiagen, Dusseldorf, Germany). The PCR products were sequenced by Sangon Biotech Co. Ltd. (Shanghai, China) and blasted in the NCBI gene bank. A phylogenetic tree was constructed, and the species of the yeast was determined using the base sequence of the yeast. The BLAST analysis was carried out to obtain the known strains with high homology to yeast 26S rDNA sequence, and the gene sequences of the strains with high similarity were obtained from the GenBank library for the establishment of a phylogenetic tree. This yeast was named *Pichia anomola* AR_2016_ and was stored at the China Center for Type Culture Collection (No. CCTCC M2017594, http://cctcc.whu.edu.cn/).

#### Determination of Protein and Amino Acid Content in *R. mucilaginosa* TZR_2014_ Cell

Three-day cultures in the PDA liquid medium were collected, and dry matter (DM) was measured by drying to a constant mass in a forced-air oven at 95°C. The amino acids in the dry yeast were determined using a Hitachi L-8800 automatic amino acid analyser.

#### Optimization of the Fermentation Condition for Carotenoid Production by *R. mucilaginosa* TZR_2014_

The fermentation condition for carotenoid production by *R. mucilaginosa* TZR_2014_ was optimized by a three-factor and three-level (3 × 3) experimental design, denoted as L9 (3^3^). Factor A was glucose with 3, 4, or 5% glucose. Factor B was peptone at 1, 1.5, or 2, and factor C was yeast powder at 0.5, 1, or 1.5. The 10% *R. mucilaginosa* culture was inoculated into the 90% fermentation media and fermented at 28°C with shaking at 200 rpm for 96 h. After fermentation, the carotenoid content was measured according to the introduction of GB5413.35-2010 (Ministry of Agriculture and Rural Affairs, Beijing, China). The biomass of *R. mucilaginosa* was measured by the weight of *R. mucilaginosa* TZR_2014_ cells.

#### Resistance Tests of *R. mucilaginosa* TZR_2014_

The logarithmic phase *R. mucilaginosa* TZR_2014_ was centrifuged and washed twice with 0.85% saline to obtain a suspension for later use. The suspension (1 × l0^9^ CFU/ml) was immersed in a water bath at 35, 45, 55, 65, or 75°C for 15 min and then cooled rapidly. Live bacteria were counted using a dilution plate, and three repetitions were performed. The suspension (1 × l0^8^ CFU/ml) was inoculated in the YPD liquid medium containing 0, 0.1, 0.2, 0.3, 0.4, or 0.5% (w/v) pig bile salt and then cultured at 37°C for 4 h. Live bacteria were counted using a dilution plate, and three repetitions were performed. The suspension (1 × l0^7^ CFU/ml) was inoculated in artificial gastric juice (100 ml hydrochloric acid (pH = 2) containing 1 g pepsin) and then cultured at 37°C for 0, 0.5, 1, 1.5, or 2 h. Live bacteria were counted using a dilution plate, and three repetitions were performed. The suspension (1 × l0^7^ CFU/ml) was inoculated into the artificial intestinal fluid (100 ml potassium dihydrogen phosphate solution (pH 6.8) containing 1 g trypsase) and then cultured at 37°C for 0, 1, 2, 3, 4, or 5 h. Live bacteria were counted using a dilution plate, and three repetitions were performed.

### Effects on Growth and Health in Weaned Pigs fed *R. mucilaginosa* TZR_2014_

#### Strain

*R. mucilaginosa* TZR_2014_ and *C. utilis* were purchased from Deutsche Sammlung von Mikroorganismen und Zellkulturen (No. DSM 2361).

#### Animal Use and Care

Thirty 28-day-old weaned pigs (Large White × Landrace × Rongchang) were randomly divided into three groups with 10 barrows each. The piglets were fed (1) a basal diet (CON), (2) a basal diet and orally administered 1 ml 1.0 × 10^10^ CFU/ml *Candida utilis* DSM 2361 three times (*C. utilis*), and (3) a basal diet and orally administrated 1 ml 1.0 × 10^10^ CFU/ml *R. mucilaginosa* TZR_2014_ three times daily (*R. mucilaginosa*); the experiment lasted for 4 weeks. All piglets were fed a diet formulated according to the National Research Council requirements (2012). The ingredients and compositions of the basal diet are presented in Table 1. The piglets were individually kept in pens in a mechanically ventilated and temperature-controlled room (22 ± 1.2°C). Food and water were provided *ad libitum*. All experimental procedures involving pigs were approved by the License of Experimental Animals (IACAU-20160322-02) of the Animal Experimentation Ethics Committee of the Southwest University, Chongqing, China.

**Table 1:** The ingredients and nutritional levels of basal diets (DM basis, %).

**Ingredients**	**Content**	**Nutrition level**	**Content**
Corn	62.78	DE/(MJ/kg)^c^	13.81
Soybean meal	19.90	CP	16.50
Fish meal	2.30	Ca	0.73
Whey powder	6.03	CF	2.60
Wheat Bran	5.00	AP	0.37
Soybean oil	0.85	Lys	1.27
Limestone	0.77	Met	0.37
Salt	0.30	Thr	0.75
CaHPO_4_	0.70	Trp	0.22
Sweetener	0.06		
Antioxidant	0.02		
Choline chloride	0.08		
Vitamin premix^a^	0.08		
Trace mineral premix^b^	0.30		
Threonine (Thr)	0.09		
Lysine Hydrochloride (Lys)	0.31		
Methionine (Met)	0.09		
Tryptophan (Trp)	0.34		
Total	100.00		

*AP, available phosphorus*.

*^a^Vitamin premix provided the following per kg of the diet: vitamin A 2 017 IU, vitamin D 208 IU, vitamin E 14 IU, vitamin K 0.49 mg, pantothenic acid 10.1 mg, riboflavin 3.4 mg, folic acid 0.29 mg, nicotinic acid 29.1 mg, thiamine 1.1 mg, vitamin B_6_ 5.7 mg, biotin 0.06 mg, vitamin B_12_ 0.017 mg*.

*^b^Trace mineral premix provided the following per kg of the diet: ZnSO_4_·7H_2_O 268 mg, FeSO_4_·7H_2_O 323.33 mg, MnSO_4_·H_2_O 11.54 mg, CuSO_4_·5H_2_O 22.86 mg, KI 14.21 mg, and Na_2_SeO_3_ 28.37 mg*.

*^c^DE was a calculated value, whereas the others were measured*.

#### Measurements and Sampling

The experimental period lasted for 4 weeks. Piglets were weighed on day 29 before the morning feed. The feed intake was recorded daily throughout the study period. Prior to the morning feed on day 29, a 10-ml blood sample of five piglets selected from each group was collected. The blood sample was undisturbed for 60 min and then centrifuged at 3,500 × *g* for 10 min at 4°C to harvest the serum. Serum was stored at −20°C for biochemical analysis and ELISA. After blood sampling, five piglets selected from each group were anesthetized with an intravenous injection of sodium pentobarbital (50 mg/kg body weight (BW)) and bled by exsanguination. The jejunal mucosa was rinsed with cold saline, scraped gently with a scalpel blade, and collected. The harvested jejunal mucosa was immediately frozen in liquid N_2_ and stored at −80°C.

#### Biochemical Analysis

The jejunal mucosa sample (1 g) was homogenized in 9 ml of 0.9% NaCl with a polytron (Brinkmann Instruments Inc., Westbury, NY, USA) and centrifuged at 6,500 × *g* for 20 min at 4°C. The supernatant was collected and stored at −80°C for later analysis. The concentrations of alkaline phosphatase (ALP), malondialdehyde (MDA), lysozyme, glutathione peroxidase (GSH-Px), nitrogen oxide synthase (NOS), total superoxide dismutase (T-SOD), and total antioxidant content (T-AOC) in the serum were determined by colorimetric methods using AKP, MDA, lysozyme, GSH-Px, NOS, T-SOD, and T-AOC reagent kits (Nanjing Jianchen Institute of Bioengineering, Nanjing, Jiangsu, China), according to the manufacturer's instructions. The concentrations of pepsin, lipase, and amylase in the jejunum were determined by colorimetric methods using pepsin, lipase, and amylase reagent kits (Nanjing Jianchen Institute of Bioengineering, Nanjing, Jiangsu, China), according to the manufacturer's instructions.

#### Hematoxylin and Eosin (H and E) Staining

The morphology of the jejunum and ileum was analyzed according to the hematoxylin and eosin (H&E) staining method described by Wang et al. (2009). The sliced samples were viewed under an optical microscope (Carl Zeiss Inc., Oberkochen, Germany). Digital images were captured using a color video camera Sony 3CCD-VX3 camcorder (Sony Corporation, Tokyo, Japan). Villus height and crypt depth were measured using the image analysis software (Intronic GmbH & Co., Rothenstein, Berlin, Germany).

#### Diarrhea Rate

Piglet fecal conditions were observed at 8:00 every day. Strip or granular feces were assessed to be 0; soft stool feces were assessed to be 1; thick and water feces were assessed to be 2; and liquid, unformed, and water feces were assessed to be 3. The formula of diarrhea rate was given as follows: diarrhea rate = fecal stool score/total number of test piglets × test days. The higher the value, the more severe the diarrhea.

#### Analysis of the MiSeq Data

Samples of caecum contents collected on day 28 were directly used for DNA extraction according to a bead-beating method using a minibead beater, followed by the introduction of the Power Fecal DNA Isolation Kit (Mobio), and DNA was quantified using a Nanodrop spectrophotometre (Nyxor Biotech), followed by staining with the Quant-it Pico Green dsDNA Kit (Invitrogen Ltd.). PCR amplification of the V4 region of bacterial 16S rDNA was performed using universal primers 515F (5'-GTGCCAGCMGCCGCGGTAA-3') and 806R (5'-GGACTACHVGGGTWTCTAAT-3') incorporating the FLX titanium adapters and a sample bar code sequence; the cycling parameters were as follows: 5 min initial denaturation at 95°C; 25 cycles of denaturation at 95°C (30 s), annealing at 55°C (30 s), elongation at 72°C (30 s), and final extension at 72°C for 5 min. Three separate PCR reactions for each sample were pooled for the MiSeq analysis. The PCR products were separated by 1.5% agarose gel electrophoresis and purified using a QIAquick Gel Extraction Kit (Qiagen). Amplicons were quantified using a Quant-iT Pico Green dsDNA Assay Kit (Invitrogen). Equal concentrations of the amplicons were pooled for each sample. Libraries were constructed using the TruSeq DNA PCR-Free Sample Prep Kit (Illumina), and MiSeq was performed using the MiSeq Reagent Kit v2 (Illumina).

In total, 747,166 raw reads were obtained from MiSeq. All reads were based on bar codes and primer sequences. The resulting sequences were further screened and filtered for quality and length analysis. Sequences that were less than 200 nucleotides in length, contained ambiguous characters, contained over two mismatches to the primers, or contained mononucleotide repeats of over six nucleotides were removed. High-quality sequences were assigned to samples according to bar codes. Sequences with a similarity level of 97% were clustered into operational clustering of taxonomic units (OTUs) using the UPARSE algorithm 7. The representative sequence from OTUs at a 0.03 distance was obtained and classified using the RDP Bayesian classifier. Any sequence annotations for chloroplast, mitochondrial, or archaeal OTUs not identified to be of bacterial origin were excluded from further analyses. We calculated diversity, bias-corrected Chao1 richness estimator, Faith's phylogenetic diversity (PD), and Shannon and Simpson diversity indices. All analyses were performed using the MOTHUR program (v1.24) (http://www.mothur.org). Based on the above results at the community composition (compositional), development, and system (phylogenetic) levels, the species-level differences in species analysis and correlation analysis, alpha diversity, and community structure diversity analysis of the samples were analyzed using the QIIME, Mothur, and R software, respectively.

### Data Calculation and Statistical Analysis

All data are presented as mean ± SEM. The data were subjected to a one-way analysis of variance using the general linear model (GLM) procedure in the SAS statistical software (SAS Institute, Inc. Cary, NC, USA) according to a completely randomized factorial design. The SNK test was performed to identify differences between the groups. Statistical significance was set at *P* < 0.05.

## Results

### Characteristics of *R. mucilaginosa* TZR_2014_

**Figure 1:**
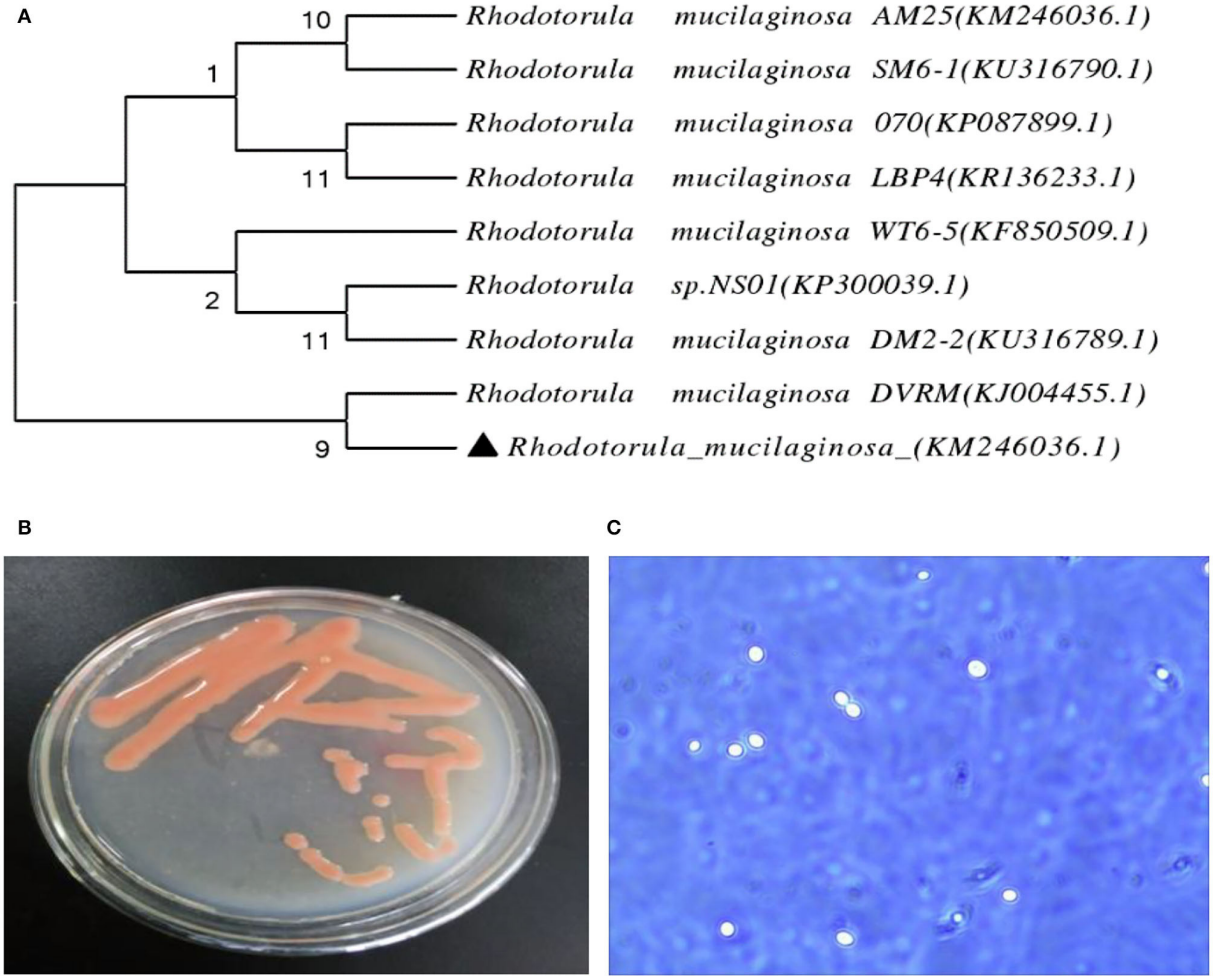
The phylogenetic tree of *Rhodotorula mucilaginosa* TZR_2014_ **(A)**, the visual colony morphology **(B)**, and the cell morphology of *Rhodotorula mucilaginosa* TZR_2014_ **(C)**.

Identification by physiological biochemical methods and 26S rDNA D1/D2 sequence analysis revealed that the red yeast was *R. mucilaginosa* (Figure 1A). This strain was named TZR_2014_ and was stored at the China Center for Type Culture Collection (CCTCC M2015574, CCTCC, Wuhan, China) on 24 September 2015. The conserved internal transcribed sequence (ITS) region of *R. mucilaginosa* TZR_2014_ was submitted to sequencing data from NCBI GenBank (accession number MT940480). The colonies of *R. mucilaginosa* TZR_2014_ were round, orange-red, bulge, colloid sticky, with neat edges and smooth surfaces (Figure 1B). The cells of *R. mucilaginosa* TZR_2014_ were round and budded (Figure 1C). As shown in Table 2, *R. mucilaginosa* TZR_2014_ uses galactose, sucrose, raffinose, sorbitol, melezitose, trehalose, mannitol, xylose, ribose, glycerol, and arabinose as carbon sources for fermentation. *R. mucilaginosa* TZR_2014_ contained 17 amino acids. The contents of aspartic acid, glutamic acid, and alanine were relatively high, and the crude protein content was 37.8% ± 0.52 (Table 3).

**Table 2:** Carbon source assimilation of *Rhodotorula mucilaginosa* TZR_2014_.

**Carbon source assimilation**	**Results**	**Carbon source assimilation**	**Results**
Galactose	+	Acetamide	_
Sucrose	+	Mannitol	+
Cellobiose	_	Rhamnose	_
Raffinose	+	Melibiose	_
Sorbitol	+	Xylose	+
Melezitose	+	Ribose	+
Inositol	_	Glycerol	+
Trehalose	+	Arabinose	+

“*+” said positive; “-” means negative*.

**Table 3:** Protein content and amino acids composition of *Rhodotorula mucilaginosa* TZR_2014_ (DM basis, %).

**Items**	**Content**	**Items**	**Content**	**Items**	**Content**
Aspartic acid	2.03 ± 0.06	Cysteine	0.19 ± 0.01	Phenylalanine	1.05 ± 0.04
Threonine	1.14 ± 0.04	Valine	1.25 ± 0.03	Lysine	1.26 ± 0.01
Serine	1.14 ± 0.04	Methionine	0.39 ± 0.03	Histidine	0.58 ± 0.01
Glutamic acid	2.29 ± 0.05	Isoleucine	1.05 ± 0.04	Arginine	1.74 ± 0.04
Glycine	1.33 ± 0.03	Leucine	1.77 ± 0.02	Proline	1.16 ± 0.07
Alanine	2.02 ± 0.05	Tyrosine	0.65 ± 0.02	Crude protein	37.8 ± 0.52

### Resistance of *R. mucilaginosa* TZR_2014_

**Figure 2:**
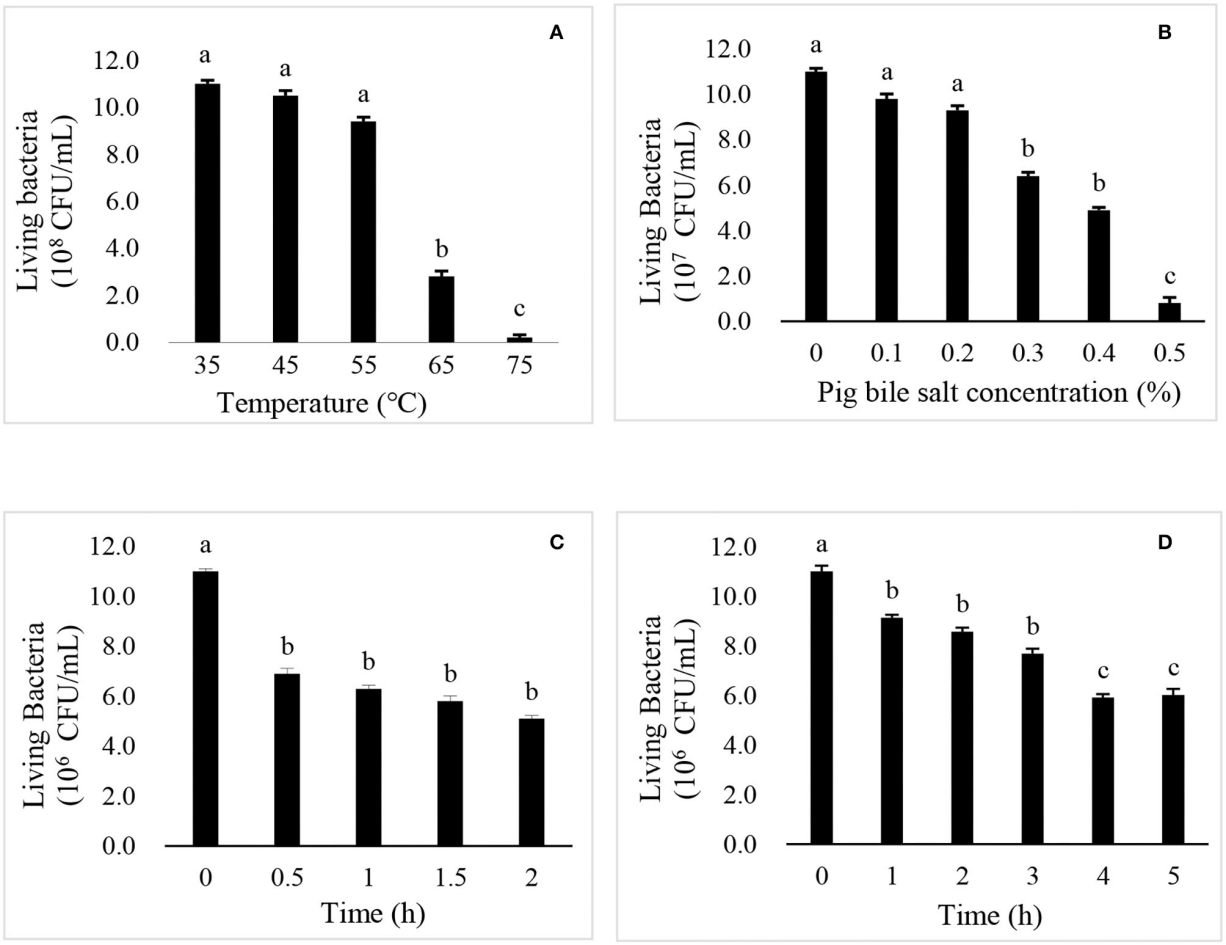
The effects of temperature **(A)**, pig bile salts **(B)**, artificial gastric juice **(C)**, and artificial intestinal fluid **(D)** on the growth of *R. mucilaginosa* TZR_2014_.

As shown in Figure 2A, there was no significant difference in the live cell number of *R. mucilaginosa* TZR_2014_ cultured at 35, 45, and 55°C. The number of live cells of *R. mucilaginosa* TZR_2014_ cultured at 65°C sharply decreased. *R. mucilaginosa* TZR_2014_ cultured at 75°C barely survived. As shown in Figure 2B, *R. mucilaginosa* TZR_2014_ grew well in a medium containing less than 0.2% pig bile salt. There was no significant difference in the live cell number of *R. mucilaginosa* TZR_2014_ in media containing 0, 0.1, and 0.2% pig bile salt (*P* > 0.05). The live cell number of *R. mucilaginosa* TZR_2014_ in the medium with 0.3% and 0.4% pig bile salt decreased (*P* < 0.05). *R. mucilaginosa* TZR_2014_ in medium with more than 0.5% pig bile salt hardly grew.

As shown in Figure 2C, the live cell number of *R. mucilaginosa* TZR_2014_ in artificial gastric juice for 0.5 h significantly decreased (*P* < 0.05). There was no significant difference in the live cell number of *R. mucilaginosa* TZR_2014_ in artificial gastric juice at 0.5, 1, 1.5, and 2 h (*P* > 0.05). As shown in Figure 2D, the live cell number of *R. mucilaginosa* TZR_2014_ in artificial intestinal fluid for 0.5 h significantly decreased (*P* < 0.05). There was no significant difference in the number of live cells of *R. mucilaginosa* TZR_2014_ in artificial intestinal fluid at 1, 2, and 3 h (*P* > 0.05). The number of *R. mucilaginosa* TZR_2014_ cells in artificial intestinal fluid for 4 h significantly decreased (*P* < 0.05). There was no significant difference in the live cell number of *R. mucilaginosa* TZR_2014_ cultured in artificial intestinal fluid for 4 and 5 h (*P* > 0.05).

### Fermentation Optimisation of *R. mucilaginosa* TZR_2014_ and Biomass and Carotenoid Production Conditions

As shown in Table 4, the biomass (25.48 g/L), carotenoid content (201.57 ± 54.98 μg/g), and carotenoid yield (15.44 ± 1.40 mg/L) of *R. mucilaginosa* TZR_2014_ reached the maximum when the contents of each component of the optimized fermentation medium were 5% glucose, 1% peptone, and 1.5% yeast powder.

**Table 4:** The result of the fermentation optimisation of *Rhodotorula mucilaginosa* TZR_2014_.

**Test number**	**A**	**B**	**C**	**Biomass (g/L)**	**Carotenoid content (μg/g)**	**Carotenoid yield (mg/L)**
1	I	I	I	16.91	164.34 ± 26.12	2.74 ± 0.47
2	I	II	II	18.02	186.03 ± 26.14	3.35 ± 0.46
3	I	III	III	20.26	161.78 ± 15.75	3.27 ± 0.32
4	II	I	II	21.30	181.49 ± 13.26	3.86 ± 0.28
5	II	II	III	23.41	193.10 ± 48.02	4.50 ± 1.12
6	II	III	I	21.60	185.58 ± 24.77	4.00 ± 0.53
7	III	I	III	25.48	201.57 ± 54.98	15.44 ± 1.40
8	III	II	I	23.23	167.35 ± 12.99	11.83 ± 0.29
9	III	III	II	24.64	180.04 ± 12.93	4.43 ± 0.32
I/3				18.39	21.23	20.68
II/3				22.10	21.65	21.32
III/3				24.55	22.17	23.05
R				6.16	0.94	2.37
I/3				170.71	182.46	172.42
II/3				186.72	182.16	182.52
III/3				175.8	175.80	185.48
R				16.01	6.66	13.06
I/3				3.12	7.34	6.19
II/3				4.12	6.56	3.88
III/3				10.56	3.90	7.73
R				7.44	3.44	3.85

### Effects of Orally Administered *C. utilis* and *R. mucilaginosa* TZR_2014_ on the Growth Performance and Diarrhea Incidence in Weaned Piglets

The final BW and average daily gain of pigs differed among the three groups (*P* < 0.01) (Table 5); that is, it was highest in the *R. mucilaginosa* group and lowest in the CON group (*P* < 0.05). Feed/gain and diarrhea rates of pigs differed among the three groups (*P* < 0.01) (Table 5); that is, they were the lowest in the *R. mucilaginosa* group and highest in the CON group (*P* < 0.05). Pigs orally administered *C. utilis* and *R. mucilaginosa* had a higher average daily food intake (*P* < 0.05) than pigs fed the basal diet. The average daily food intake did not differ between *C. utilis* and *R. mucilaginosa* groups (*P* > 0.05).

**Table 5:** Effects of *C. utilis* DSM 2361 and *R. mucilaginosa* TZR_2014_ on the growth performance and diarrhea incidence in weaned piglets (*n* = 10).

**Items**	**Treatments** ^**a**^			**SEM^**b**^**	***P*-value**
	**CON**	***C. utilis***	***R. mucilaginosa***		
Intial BW (kg)	7.23	7.25	7.20	0.02	1.00
Final BW (kg)	11.9^C^	13.5^B^	14.5^A^	0.09	0.01
Average daily gain (ADG) (g/d)	166^C^	223^B^	259^A^	3.56	<0.01
Average daily feed intake (ADFI) (g/d)	382^B^	438^A^	449^A^	4.51	0.036
Feed/gain (F/G)	2.3^A^	1.96^B^	1.73^C^	0.03	<0.01
Diarrhea rate (%)	17.7^A^	17.0^A^	14.3^B^	0.08	0.01

*^a^CON, piglets were fed a corn-and a soybean-based basal diet; C. utilis, piglets were fed a soybean-based basal diet and orally administrated 1 ml 1.0 × 10^10^ CFU/ml C. utilis DSM 2361 three times; R. mucilaginosa, piglets were fed a soybean-based basal diet and daily orally administrated 1 mL 1.0 × 10^10^ CFU/ml R. mucilaginosa TZR_2014_ three times daily*.

*^b^Standard error mean.*

*^ABC^Values in the same row with different superscript letters indicate significant differences (P <0.05)*.

### Effects of Orally Administered *C. utilis* and *R. mucilaginosa* TZR_2014_ on Intestinal Morphological Structure in Weaned Piglets

As shown in Table 6, there was no significant difference in jejunal or ileal length among the three groups (*P* > 0.05). Compared with the piglets in the CON and *C. utilis* groups, those in the orally administered *R. mucilaginosa* group had increased jejunum weight, ileum weight, and ileum villus height (*P* < 0.05). The jejunum weight, ileum weight, and ileum villus height did not differ between *C. utilis* and *R. mucilaginosa* groups (*P* > 0.05). Compared with the piglets in the CON group, those in the orally administered *R. mucilaginosa* and *C. utilis* groups had decreased ileal crypt depth. The ileum crypt depth did not differ between *C. utilis* and *R. mucilaginosa* groups (*P* > 0.05).

**Table 6:** Effects of *C. utilis* DSM 2361 and *R. mucilaginosa* TZR_2014_ on intestinal structure in weaned piglets (*n* = 5).

**Items**	**Treatments** ^**a**^			**SEM^**b**^**	***P*-value**
	**CON**	***C. utilis***	***R. mucilaginosa***		
Jejunum length (cm)	344	363	420	32.5	0.28
Ileum length (cm)	533	561	580	25.5	0.45
Jejunum weight (g)	162^B^	205^B^	310^A^	27.6	0.03
Ileum weight (g)	223^B^	294^B^	413^A^	45.6	0.01
Ileum villus height (μm)	221^B^	236^B^	276^A^	50.1	0.02
Ileum crypt depth (μm)	201^A^	154^B^	172^B^	30.2	0.01

*^a^CON, piglets were fed a corn-and a soybean-based basal diet; C. utilis, piglets were fed a soybean-based basal diet and orally administrated 1 ml 1.0 × 10^10^ CFU/ml C. utilis DSM 2361 three times; R. mucilaginosa, piglets were fed a soybean-based basal diet and daily orally administrated 1 ml 1.0 × 10^10^ CFU/ml R. mucilaginosa TZR_2014_ three times daily*.

*^b^Standard error mean.*

*^ABC^Values in the same row with different superscript letters indicate significant differences (P <0.05)*.

**Figure 3:**
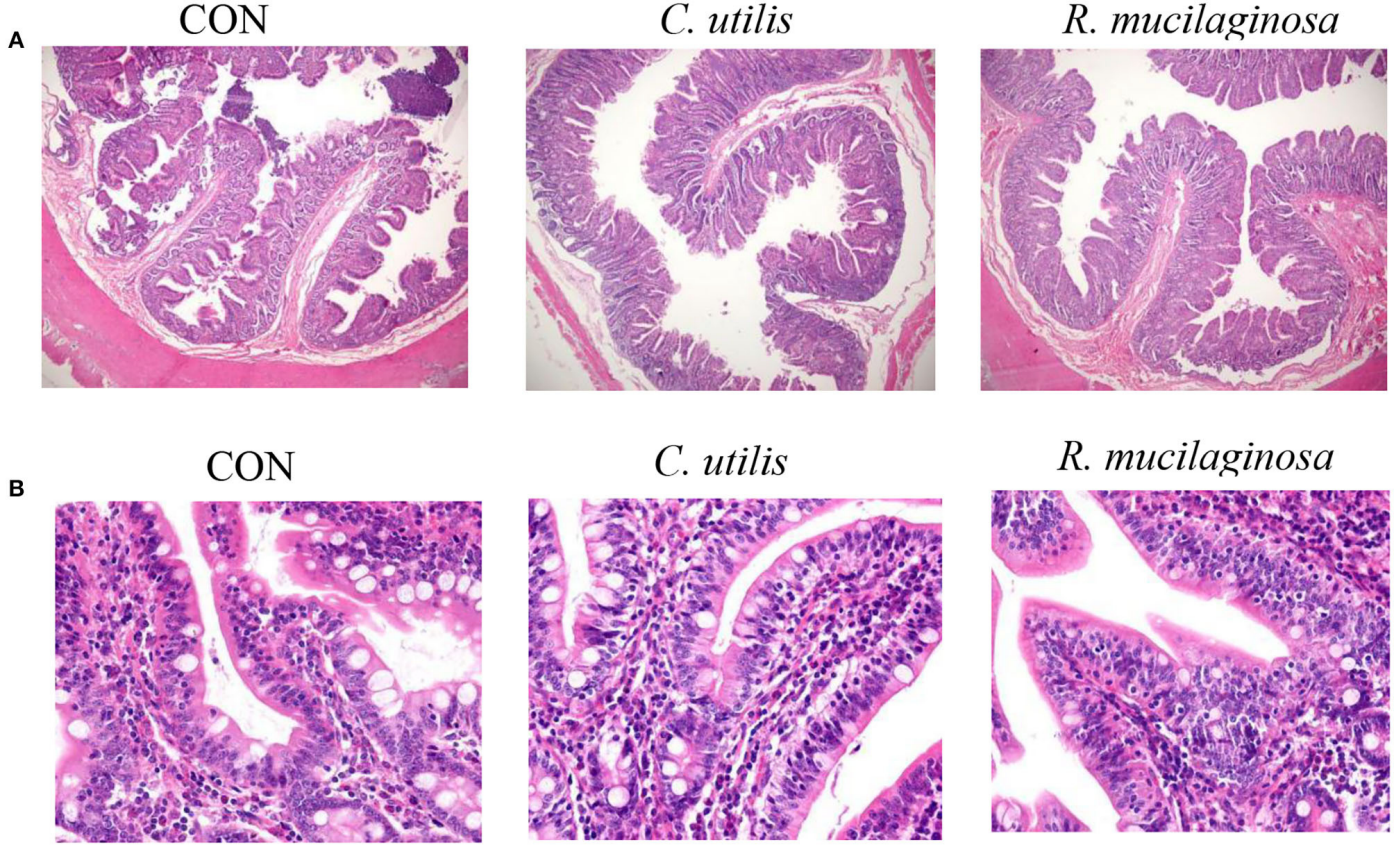
Effects of *C. utilis* DSM 2361 and *R. mucilaginosa* on ileum morphology by magnification 40 × **(A)** and 400 × **(B)** in weaned piglets. CON, piglets were fed a corn-and a soybean-based basal diet; *C. utilis*, piglets were fed a soybean-based basal diet and orally administrated 1 ml 1.0 × 10^10^ CFU/ml *C. utilis* DSM 2361 three times; *R. mucilaginosa*, piglets were fed a soybean-based basal diet and daily orally administrated 1 ml 1.0 × 10^10^ CFU/ml *R. mucilaginosa* TZR_2014_ three times daily.

According to H&E-stained sections (Figure 3), some villi in the CON group were broken and missing. The villi in the *C. utilis* and *R. mucilaginosa* groups were clear and neatly arranged. Compared with that in the CON group, the villi in the *C. utilis* and *R. mucilaginosa* groups were more complete.

### Effects of Orally Administered *C. utilis* and *R. mucilaginosa* TZR_2014_ on Blood Biochemical Indicators in Weaned Piglets

As shown in Table 7, there were no significant differences in serum ALP, NOS, or lysozyme concentrations among the three groups (*P* > 0.05). Compared with the piglets in the CON group, those in the orally administered *R. mucilaginosa* and *C. utilis* groups had increased serum T-AOC concentration. Serum T-AOC concentrations did not differ between *C. utilis* and *R. mucilaginosa* groups (*P* > 0.05). Serum T-SOD and GSH-Px concentrations differed among the three groups (*P* < 0.01) and were the highest in the *R. mucilaginosa* group and lowest in the CON group (*P* < 0.05). Serum MDA differed among the three groups (*P* < 0.01); that is, it was highest in the CON group and lowest in the *C. utilis* group (*P* < 0.05).

**Table 7:** Effects of *C. utilis* DSM 2361 and *R. mucilaginosa* TZR_2014_ on serum blood biochemical indicators in weaned piglets (*n* = 5).

**Items**	**Treatments** ^**a**^			**SEM^**b**^**	***P*-value**
	**CON**	***C. utilis***	***R. mucilaginosa***		
Alkaline phosphatase (ALP) (King unit/100 mL)	16.9	17.1	17.4	0.1	0.21
Lysozyme (U/mL)	12.3	14.2	14.1	0.5	0.45
Nitricoxide synthase (NOS) (U/mL)	13.3	14.1	13.9	0.2	0.52
Total antioxidative capacity (T-AOC) (U/mL)	1.3^B^	1.6^A^	1.6^A^	0.02	<0.01
Total superoxide dismutase (T-SOD) (U/mg prot)	52.5^C^	58.2^B^	60.7^A^	0.77	0.03
Glutathione peroxidase (GSH-Px) (U/mg prot)	470^C^	516^B^	768^A^	4.5	<0.01
Malondialdehyde (MDA) (nmol/mL)	4.9^A^	2.3^C^	2.6^B^	0.07	<0.01

*^a^CON, piglets were fed a corn-and a soybean-based basal diet; C. utilis, piglets were fed a soybean-based basal diet and orally administrated 1 ml 1.0 × 10^10^ CFU/ml C. utilis DSM 2361 three times; R. mucilaginosa, piglets were fed a soybean-based basal diet and daily orally administrated 1 ml 1.0 × 10^10^ CFU/mL R. mucilaginosa TZR_2014_ three times daily*.

*^b^Standard error mean.*

*^ABC^Values in the same row with different superscript letters indicate significant differences (P <0.05)*.

### Effects of Orally Administered *C. utilis* and *R. mucilaginosa* TZR_2014_ on Digestive Enzyme Activities of Jejunal Contents and pH of Gastrointestinal Tract in Weaned Piglets

As shown in Table 8, compared with the piglets in the CON group, those in the orally administered *R. mucilaginosa* and *C. utilis* groups had increased pepsin activity in jejunal contents. The pepsin activity of jejunal contents did not differ between *C. utilis* and *R. mucilaginosa* groups (*P* > 0.05). There was no significant difference in the amylase activity of jejunal contents or stomach pH among the three groups (*P* > 0.05). The lipase activity of the jejunal contents differed among the three groups (*P* < 0.01); that is, the value was the highest in the *R. mucilaginosa* group and lowest in the CON group (*P* < 0.05). Compared with the piglets in the CON and *C. utilis* groups, those in the orally administered *R. mucilaginosa* group had increased jejunum pH. Jejunum pH values did not differ between the CON and *C. utilis* groups (*P* > 0.05). Compared with the piglets in the CON group, those in the orally administered *R. mucilaginosa* and *C. utilis* groups had increased pH in the ileum, colon, and caecum. The pH values of the ileum, colon, and caecum did not differ significantly between the *R. mucilaginosa* and *C. utilis* groups (*P* > 0.05).

**Table 8:** Effects of *C. utilis* DSM 2361 and *R. mucilaginosa* TZR_2014_ on digestive enzyme activity of jejunal contents and pH of gastrointestinal tract in weaned piglets (*n* = 5).

**Items**	**Treatments** ^**a**^			**SEM^**b**^**	***P*-value**
	**CON**	***C. utilis***	***R. mucilaginosa***		
Pepsin (U/mg prot)	27.0^B^	30.5^A^	31.4^A^	0.46	0.032
Amylase (U/mg prot)	0.047	0.070	0.193	0.08	0.41
Lipase (U/mg prot)	144^C^	346^B^	388^A^	7.17	<0.01
Stomach pH	3.5	3.0	3.3	0.09	0.058
Jejunum pH	5.9^A^	5.9^A^	5.5^B^	0.04	<0.01
Ileum pH	6.7^A^	6.3^B^	6.3^B^	0.08	0.013
Colon pH	6.3^A^	6.2^B^	5.9^B^	0.11	0.048
Cecum pH	6.2^A^	5.8^B^	5.8^B^	0.06	<0.01

^a^*CON, piglets were fed a corn-and a soybean-based basal diet; C. utilis, piglets were fed a soybean-based basal diet and orally administrated 1 ml 1.0 × 10^10^ CFU/ml C. utilis DSM 2361 three times; R. mucilaginosa, piglets were fed a soybean-based basal diet and daily orally administrated 1 ml 1.0 × 10^10^ CFU/mL R. mucilaginosa TZR_2014_ three times daily*.

*^b^Standard error mean.*

*^ABC^Values in the same row with different superscript letters indicate significant differences (P <0.05)*.

### Analysis of Colon Microbial Ecology in Weaned Piglets Orally Administered *R. mucilaginosa* TZR_2014_

As shown in Table 9, the dominant bacteria in the colonic contents of the three groups were mainly Proteobacteria, Bacteriodetes, and Firmicutes, and the total number of microbes of the three types of bacteria reached more than 90%. Compared with the piglets in the CON and *C. utilis* groups, piglets in the orally administered *R. mucilaginosa* group showed an increasing trend in diversity, Chao1, Shannon, and Faith_ID indices (*P* > 0.05).

**Table 9:** Effects of *C. utilis* DSM 2361 and *R. mucilaginosa* TZR_2014_ on alpha diversity and of phylum and genus level microbial composition in cecum of weaned piglets (*n* = 5).

**Items**	**Treaments** ^**a**^			**SEM^**b**^**	***P-value***
	**CON**	***C.utilis***	***R.mucilaginosa***		
Diversity	845	967	1,056	55.4	0.09
Chao1	1,227	1,401	1,573	139.6	0.19
Simpson	0.96	0.98	0.98	0.11	0.33
Shannon	4.8	5.3	5.5	0.17	0.10
Faith_PD	70.9	77.1	79.4	2.11	0.07
**Phylum (%)**					
*Bacteroidetes*	61.9^A^	60.1^A^	52.3^B^	1.43	0.007
*Firmicutes*	25.2^B^	24.0^B^	31.1^A^	1.10	0.008
*Proteobacteria*	11.5^A^	11.2^A^	6.4^B^	0.77	0.006
*Verrucomicrobia*	0.03^C^	1.0^B^	6.4^A^	0.21	<0.01
*Actinobacteria*	2.4^A^	1.1^B^	1.2^B^	0.14	0.001
*Spirochaetae*	0.07^B^	0.4^B^	1.8^A^	0.18	0.001
*Acidobacteria*	0.07	0.03	0.07	0.03	0.73
**Genus (%)**					
*Prevotella*	16.86^B^	18.10^B^	22.01^A^	0.905	0.016
*Prevotellaceae NK3B31 group*	2.15^C^	19.56^A^	9.16^B^	0.517	<0.01
*Bacteroidales S24-7 group*	3.2	6.13	4.85	1.282	0.337
*Ruminococcaceae*	1.43^C^	4.15^B^	8.00^A^	0.156	<0.01
*Lachnospiraceae*	2.13	4.48	6.06	0.904	0.057
*Alloprevotella*	3.19^B^	4.18^A^	4.03^A^	0.189	0.021
*Succinivibrio*	2.08^B^	4.31^A^	4.42^A^	0.157	<0.01
*Rikenellaceae* RC9 gut group	1.56^B^	2.53^B^	4.03^A^	0.326	0.005
*Roseburia*	1.39^B^	1.51^B^	3.36^A^	0.391	0.02
*Anaerovibrio*	1.01	3.03	1.78	1.137	0.492
*Clostridium sensu stricto 1*	1.41	1.1	2.31	0.783	0.557
*Bacteroides*	1.49	1.81	1.37	0.181	0.29
*Lactobacillus*	3.91^A^	0.40^B^	0.31^B^	0.61	0.009
*Parabacteroides*	0.63	2.77	1.69	0.829	0.266
*Akkermansia*	0.03^B^	1.5^A^	1.44^A^	0.174	0.002
*Megasphaera*	1.65^A^	0.24^B^	0.45^B^	0.134	0.001

^a^*CON, piglets were fed a corn-and a soybean-based basal diet; C. utilis, piglets were fed a soybean-based basal diet and orally administrated 1 ml 1.0 × 10^10^ CFU/ml C. utilis DSM 2361 three times; R. mucilaginosa, piglets were fed a soybean-based basal diet and daily orally administrated 1 ml 1.0 × 10^10^ CFU/mL R. mucilaginosa TZR_2014_ three times daily*.

*^b^Standard error mean.*

*^ABC^Values in the same row with different superscript letters indicate significant differences (P <0.05)*.

The relative abundances of seven phyla are shown in Table 9. Compared with that in the CON and *C. utilis* groups, the relative abundance of Bacteroidetes and Proteobacteria in the orally administered *R. mucilaginosa* TZR_2014_ group decreased (*P* < 0.05). The relative abundances of Bacteroidetes and Proteobacteria did not differ between the CON and *C. utilis* groups (*P* > 0.05). Compared with that in the CON group, the relative abundance of Actinobacteria in the orally administered *R. mucilaginosa* TZR_2014_ and *C. utilis* groups decreased (*P* < 0.05). The relative abundance of Actinobacteria did not differ between *R. mucilaginosa* and *C. utilis* groups (*P* > 0.05). Compared with that in the CON and *C. utilis* groups, the relative abundance of Firmicutes and Spirochaetae in the orally administered *R. mucilaginosa* TZR_2014_ group increased (*P* < 0.05). The relative abundances of Firmicutes and Spirochaetae did not differ between CON and *C. utilis* groups (*P* > 0.05). The relative abundance of Verrucomicrobia differed among the three groups (*P* < 0.01); that is, it was the highest in the *R. mucilaginosa* group and lowest in the CON group (*P* < 0.05). There was no significant difference in the relative abundance of Acidobacteria in piglets among the groups (*P* > 0.05).

Sixteen genus groups with relative abundances greater than 1% are shown in Table 9. Compared with that in the CON and *C. utilis* groups, the relative abundance of Prevotella, Rikenellaceae RC9 gut group, and Roseburia in the orally administered *R. mucilaginosa* TZR_2014_ decreased (*P* < 0.05). The relative abundances of Prevotella, Rikenellaceae RC9 gut group, and Roseburia did not differ between the CON and *C. utilis* groups (*P* > 0.05). The relative abundance of Prevotellaceae *NK3B31 group* differed among the three groups (*P* < 0.01); that is, it was the highest in the *C. utilis* group and lowest in the CON group (*P* < 0.05). The relative abundance of Ruminococcaceae differed among the three groups (*P* < 0.01); that is, it was the highest in the *R. mucilaginosa* group and lowest in the CON group (*P* < 0.05). Compared with that in the CON group, the relative abundances of Alloprevotella, Succinivibrio, and Akkermansia in the orally administered *R. mucilaginosa* TZR_2014_ and *C. utilis* groups increased (*P* < 0.05). The relative abundance of Alloprevotella, Succinivibrio, and Akkermansia did not differ between *R. mucilaginosa* and *C. utilis* groups (*P* > 0.05). Compared with that in the CON group, the relative abundance of Lactobacillus and Megasphaera in the orally administered *R. mucilaginosa* TZR_2014_ and *C. utilis* decreased (*P* < 0.05). The relative abundances of Lactobacillus and Megasphaera did not differ between *R. mucilaginosa* and *C. utilis* groups (*P* > 0.05). There was no significant difference in the relative abundance of Bacteroidales S24-7 group, Lachnospiraceae, Anaerovibrio, Clostridium sensu stricto 1, Bacteroides, or Parabacteroides of piglets among the three groups (*P* > 0.05).

## Discussion

In this experiment, a red pigment-producing yeast strain was selected from the soil of a Chongqing orchard. The colonies it formed were orange-red, and the yield of carotenoids was very high. Through identification of its colony characteristics, individual morphology, and physiological and biochemical characteristics, it was determined to be a species of *Rhodotorula*. After further analysis of the 26S rDNA D1/D2 sequence, it was identified as *R. mucilaginosa*, which contains 17 amino acids and can utilize a variety of carbon sources. Through fermentation optimisation of *R. mucilaginosa* TZR_2014_, we found that the ideal composition of the fermentation medium was 5% glucose, 1% peptone, and 1.5% yeast powder. In addition, the above results show that *R. mucilaginosa* TZR_2014_ has good tolerance to simulated gastric acid and porcine bile salt environments, indicating that *R. mucilaginosa* TZR_2014_ can be added as an additive to animal diets. This is similar to the results of Hamidi et al. (2020), who reported that *R. mucilaginosa* has high biocompatibility and low cytotoxicity.

In this study, *R. mucilaginosa* TZR_2014_ had a significant positive effect on the growth and performance of weaned piglets. The specific manifestation was that it significantly increased average daily gain and average daily feed intake in weaned piglets and significantly reduced feed/gain and diarrhea rates. The growth-promoting effect of *R. mucilaginosa* TZR_2014_ may be related to its effects on digestive enzymes, intestinal pH, and intestinal morphology. Studies have shown that feeding live yeast can promote the growth and development of fishes, increase the activity of pancreatic amylase, lipase, and protease (Tovar-RamiRez et al., 2004), and improve protein utilization and feed conversion rates (Lara-Flores et al., 2003). In addition, the metabolites of *R. mucilaginosa* TZR_2014_ include fatty acids, which can lower the pH of the intestinal tract. In this experiment, adding *R. mucilaginosa* TZR_2014_ to the diet significantly increased pepsin and lipase activities in the jejunum of weaned piglets and significantly reduced the pH value of the jejunum, ileum, colon, and caecum, similar to the above results. In terms of intestinal morphology, the ileal villus height and crypt depth of the *R. mucilaginosa* and *C. utilis* groups were greater than those of the CON group. The small intestine plays a major role in the digestion, absorption, and transport of nutrients and is the body's defense against harmful substances. This protective barrier against pathogenic microorganisms and good, intact intestinal morphology is important guarantees for maintaining the healthy growth of the body (El Aidy et al., 2015). The addition of *R. mucilaginosa* TZR_2014_ to the diet may alleviate the shortage of digestive enzymes and damage to intestinal morphology caused by weaning stress and improve feed utilization and growth performance.

Based on the serum biochemical indicators of weaned piglets, both the *R. mucilaginosa* and *C. utilis* groups showed strong antioxidant capacities, and the T-AOC, T-SOD, GSH-PX, and MDA indicators were better than those of the CON group. This may be due to the unique extracellular polysaccharide (EPS) of yeast. EPS may contain mannose, glucose, galactose, xylose, fucose, and rhamnose residues as the main chain or branch components (Ustyuzhanina et al., 2018). The EPS of *R. mucilaginosa* (composed of galactose, arabinose, glucose, and mannose with molar ratios of 63.1:0.2:18.3:18.3, respectively) has a strong free-radical scavenging ability [2,2-Diphenyl-1-picrylhydrazyl (DPPH) and 2, 2'-azino-bis(3-ethylbenzothiazoline-6-sulfonic acid) (ABTS)] and anti-tumor activity (Ma et al., 2018). Some researchers isolated linear mannan with ß-1,3 and ß-1,4 bonds produced by *R. mucilaginosa* and found that it can increase cholinesterase, α-amylase, aldolase, and red blood cell catalase in the liver and serum (Rahbar Saadat et al., 2021). The glucomannan produced by *C. utilis* is composed of D-mannose and D-glucose, which contain α-1,3, α-1,4, and α-1,6 glycosidic bonds and have strong antioxidant activity (Van Bogaert et al., 2009). Some *in vitro* experiments have shown that the isolated EPS is not cytotoxic to normal cell models, while it does exert antioxidant activity (Hamidi et al., 2020). Notably, *R. mucilaginosa* TZR_2014_ performed better than *C. utilis* in antioxidant indicators. This may be related to the unique metabolite of *R. mucilaginosa* TZR_2014_, carotenoids. Carotenoids have a strong effect on the antioxidant and immune systems. Experiments have shown that carotenoids can improve disease resistance, antioxidant capacity, and growth performance of farmed fish without causing other cytotoxicities or side effects (Nakano and Wiegertjes, 2020). Carotenoids can increase not only the activity of antioxidant enzymes (SOD and GPX) and endogenous antioxidants (GSH) but also the antioxidant capacity of cells by regenerating α-tocopherol and ascorbic acid from their corresponding free radical forms and cooperating with other antioxidants to protect lipoproteins from oxidation and reduce the body's oxidative stress (Nishida, 2012; Pisoschi and Pop, 2015; Lim et al., 2018). In addition, carotenoids can also inhibit the NF-κB signaling pathway (inflammatory response activation) and activate the Nrf2 signaling pathway (defense against ROS-induced cellular oxidative stress; Ambati et al., 2014; Gammone et al., 2015; Pisoschi and Pop, 2015; Ribeiro et al., 2018). Therefore, *R. mucilaginosa* TZR_2014_ is a promising feed additive for alleviating oxidative stress in weaned piglets.

Because the digestive and immune systems of weaned piglets have not yet fully developed, coupled with changes in diet, the number and structure of the intestinal flora have undergone great changes, which can easily lead to the proliferation of harmful bacteria and diarrhea. Studies have shown that mannan and β-glucan in the cell wall of yeast can adsorb intestinal pathogens and toxic metabolites. Their structure is similar to that of mannose residues on the surface of the intestinal epithelial cells. Mannose residues are the binding sites between pathogenic microorganisms and the intestinal tract. This yeast structure can competitively bind to pathogenic microorganisms and reduce the adhesion of these microorganisms in the intestinal tract. Because yeast is a passing visitor in the intestines, the yeast/pathogen complex can be quickly excreted from the digestive tract (Fuller, 1989; Spring et al., 2000; Li et al., 2005). In this study, orally administered *R. mucilaginosa* TZR_2014_ significantly reduced the relative abundance of Proteobacteria and Megasphaera. Proteobacteria contains many common pathogenic bacteria, such as *Escherichia coli, Salmonella*, and *Helicobacter pylori*, and harmful spoilage bacteria such as *Megasphaera*. These results were consistent with the above conclusions. In addition, orally administered *R. mucilaginosa* TZR_2014_ significantly increased the relative abundances of Prevotella, Ruminococcaceae, Succinivibrio, Rikenellaceae RC9 gut group, and Roseburia in this experiment. Prevotella is the dominant flora in the intestinal tract of weaned piglets. It can promote glucose metabolism and break down complex carbohydrates, such as polysaccharides and starch, into short-chain fatty acids (Kovatcheva-Datchary et al., 2015). Ruminococcaceae can not only decompose cellulose and carbohydrates but also synthesize secondary bile acids through 7α-dehydrogenation to reduce intestinal inflammation (Mullish et al., 2019). Succinivibrio is abundant in the rumen of high-yielding and multi-product dairy cows, and its growth is related to the activities of urease, glutamine synthase, glutamate dehydrogenase, and glutamate synthase, which can improve nitrogen utilization (Hailemariam et al., 2020). Studies have shown that the abundance of the Rikenellaceae RC9 gut group is negatively correlated with diarrhea rate and positively correlated with nutrient digestibility, similar to the results of this experiment (Tian et al., 2020). Roseburia is a saccharolytic, butyrate-producing bacterium from human feces that can decompose a variety of sugars (e.g., xylose, galactose, raffinose, maltose, cellobiose, sucrose, and starch), and also affects the activities of β-glucuronidase and β-glucosidase in *in vitro* experiments, which is conducive to improve feed utilization (Machiels et al., 2014). The number and structure of the intestinal flora are closely related to the host's nutrition and health. *R. mucilaginosa* TZR_2014_ can optimize the structure of the intestinal flora, inhibit the reproduction of harmful bacteria, and promote the utilization of nutrients and body health.

In general, this study found that *R. mucilaginosa* TZR_2014_ is safe and effective as an additive. Adding *R. mucilaginosa* TZR_2014_ to the diet can improve the growth performance of weaned piglets, promote the development of intestinal morphology, increase the activity of digestive enzymes, enhance antioxidant capacity, resist the invasion of harmful bacteria, and reduce the rate of diarrhea. However, before applying *R. mucilaginosa* TZR_2014_ to production practice, more experimental studies are needed to explore the probiotic mechanism of *R. mucilaginosa*, the best addition amount and form, and the possible side effects.

## Conclusion

*R. mucilaginosa* was screened using traditional taxonomic and molecular biological classifications and identification methods and was named TZR_2014_. *R. mucilaginosa* TZR_2014_ has a tolerance to gastric acid and bile salt environments. Oral administration of *R. mucilaginosa* TZR_2014_ improved the average daily gain and average daily feed intake of weaned piglets and reduced the feed/gain and diarrhea rates. *R. mucilaginosa* TZR_2014_ had a positive effect on improving the morphology of the small intestine, increasing the activities of digestive enzyme (pepsin and lipase), reducing gastrointestinal pH value, enhancing antioxidant performance (T-AOC, T-SOD, GSH-Px, and MDA), and improving the number and structure of intestinal flora in weaned piglets.

## Data Availability Statement

The 16sRNA sequencing raw data are available at NCBI under the accession number PRJNA827018 by web link (https://www.ncbi.nlm.nih.gov/sra/PRJNA827018). The rest of the raw data supporting the conclusions of this article will be made available by the authors without undue reservation.

## Ethics Statement

The animal study was approved by the Animal Experimentation Ethics Committee of Southwest University, Chongqing, China (IACAU-20160322-02). All piglets were raised following the guidelines described by the Animal Care Committee of Chongqing, China. In addition, efforts were made to reduce animal suffering and were carried out in compliance with the ARRIVE guidelines for reporting *in vivo* experiments in animal research.

## Author Contributions

PH and JM: investigation, data curation, methodology, formal analysis, and writing of the original draft. YZ and HD: investigation and methodology. ZS and CC: writing, review, and editing. WS: conceptualization and supervision. ZT: investigation, project administration, funding acquisition, and writing-reviewing and editing. All authors have contributed to the manuscript and approved the submitted version.

## Funding

This study was funded by the Natural Science Foundation Project of Chongqing (cstc2021jcyj-msxmX0966), the Chongqing Key Innovation Project for Overseas Students (cx2017024), the Technical Service Cooperation (M2022002, FJ20202048, and FJ2021249), and the 2021-Research Topics in Committee of Agriculture and Forestry Discipline of Chinese Society of Degree and Graduate education (2021-NL2X-YB76).

## Conflict of Interest

The authors declare that the research was conducted in the absence of any commercial or financial relationships that could be construed as a potential conflict of interest.

## Publisher's Note

All claims expressed in this article are solely those of the authors and do not necessarily represent those of their affiliated organizations, or those of the publisher, the editors and the reviewers. Any product that may be evaluated in this article, or claim that may be made by its manufacturer, is not guaranteed or endorsed by the publisher.
